# The Correlation-Base-Selection Algorithm for Diagnostic Schizophrenia Based on Blood-Based Gene Expression Signatures

**DOI:** 10.1155/2017/7860506

**Published:** 2017-02-09

**Authors:** Hang Zhang, Ziyang Xie, Yuwen Yang, Yizhen Zhao, Bao Zhang, Jing Fang

**Affiliations:** ^1^School of Mechanical Engineering, Xi'an Jiao Tong University, State Key Laboratory of Manufacturing System Engineering, Xi'an 710049, China; ^2^College of Medicine & Forensic, Health Science Center, Xi'an Jiaotong University, Xi'an 710061, China; ^3^Department of Obstetrics and Gynecology, The First Affiliated Hospital, Xi'an Jiaotong University, Xi'an 710061, China

## Abstract

Microarray analysis of gene expression is often used to diagnose different types of disease. Many studies report remarkable achievements in nervous system disease. Clinical diagnosis of schizophrenia (SCZ) still depends on doctors' experience, which is unreliable and needs to be more objective and quantified. To solve this problem, we collected whole blood gene expression data from four studies, including 152 individuals with schizophrenia (SCZ) and 138 normal controls in different regions. The correlation-based feature selection (CFS, one of the machine learning methods) algorithm was applied in this study, and 103 significantly differentially expressed genes between patients and controls, called “feature genes,” were selected; then, a model for SCZ diagnosis was built. The samples were subdivided into 10 groups, and cross-validation showed that the model we constructed achieved nearly 100% classification accuracy. Mathematical evaluation of the datasets before and after data processing proved the effectiveness of our algorithm. Feature genes were enriched in Parkinson's disease, oxidative phosphorylation, and TGF-beta signaling pathways, which were previously reported to be associated with SCZ. These results suggest that the analysis of gene expression in whole blood by our model could be a useful tool for diagnosing SCZ.

## 1. Introduction

Schizophrenia is one of the most common, severe, and heritable psychiatric disorders, with a lifetime risk of 1% in the global population [1]. Over the years, clinical diagnosis of schizophrenia has been highly dependent upon the patient's symptoms, relying mainly on self-reports, mental state examination, and clinical interviews. Due to the lack of objective laboratory tests, doctors often fail to explain the pathogenic mechanisms behind the symptoms. Therefore, patients tend to doubt the validity of the schizophrenia diagnosis [2]. Furthermore, diagnostic strategy has been widely criticized as it can sometimes lead to misdiagnosis [3].

With the development of probe and microarray techniques, many studies have been performed to investigate the relationship between gene expression and illness. Genome-wide blood transcriptome profiling coupled with network analyses provides a platform for identifying functionally relevant biological markers of disease, permitting multiscale data integration [4]. Additionally, machine learning provides a useful tool for in silico prediction of candidate biomarkers [5]. Previously, many studies on the diagnosis of cancer have been reported and show great success in providing molecular diagnostics by machine learning [6]. Such studies used tools such as Support Vector Machine or the shrunken centroid classification method to analyze microarray gene expression data to diagnose cancer [7, 8]. In recent years, there have been substantial advances in the molecular diagnosis of nervous system diseases such as Pelizaeus-Merzbacher disease [9]. Additionally, as whole blood is a relatively accessible patient sample, it is considered a valuable source of gene expression data [10]. Using blood-based gene expression and transcriptome data, a model has been constructed for diagnosis of SCZ [11].

A machine learning method called the correlation-based feature selection (CFS) algorithm was chosen to process data in our study. In this analysis, high influential genes (feature genes) are selected when they are highly associated with whether a certain patient suffers from schizophrenia and when the correlation between each selected gene is relatively small [12].

In this study, we selected and normalized 4 datasets of peripheral blood transcriptome profiling, which were then analyzed by the CFS algorithm. The feature genes were selected and used to establish a model for objective clinical diagnosis by studying the differential transcript levels in patients as compared to controls. Finally, an identification model was established that was used for objective clinical diagnosis.

## 2. Materials and Methods

**Figure 1:**
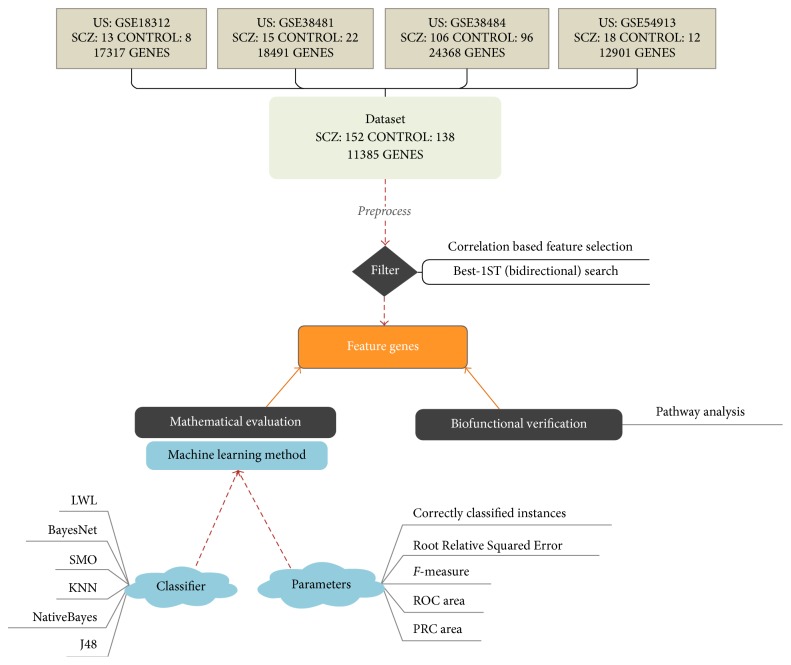
The flowchart of the diagnostic classification of schizophrenia.

The diagnostic classification of schizophrenia included four parts: the patient data, the analyzing method, feature gene evaluation, and pathway analysis. The flowchat of the diagnostic classification is illustrated in Figure 1.

### 2.1. Subjects

The purpose of this study was to analyze gene expression of SCZ patients from various regions within a large population. We searched “whole blood”, “schizophrenia” and “profile” on the GEO database of NCBI and downloaded 4 datasets (https://www.ncbi.nlm.nih.gov/gds/?term=). All datasets, including GSE18312 [13], GSE38481, and GSE38484 [14], contain RNA information of SCZ patients in America. GSE54913 [15] contains RNA information of Chinese teenagers with SCZ in China. The whole group contains 290 samples (152 SCZ patients and 138 controls). The group of adults we studied contains 134 SCZ patients (96 males and 38 females, aged 37.1 ± 11.6) and 126 controls (63 males and 63 females, aged 40 ± 13.3 years). The teenager group contains 18 SCZ patients (8 males and 10 females, aged 14.8 ± 1.7 years).  Table 1 shows that all the datasets we normalized.

To solve interplatform system heterogeneity (difference in methods used to determine the transcription group, signal extraction, and calculation process), we used a specialized data normalization algorithm to merge different datasets. We used R software package CONOR, containing the XPN (Cross-Platform Normalization) and DWD (Distance Weighted Discrimination) methods to normalize different datasets. Finally, we obtained the integrated expression spectrum matrix, containing 11385 elements (genes) and 290 columns (sample size).

### 2.2. Method Choosing

The magnitudes of gene sequencing results usually reach tens of thousands. However, the sequencing samples of SCZ vary from tens to hundreds due to limited samples. This type of data is typical of the high-dimension, low-sample-size datasets (HDLSS), which are characterized by large number of features, *p*, and a relatively small number of samples, *n*  (*p* ≫ *n*). The former study shows that HDLSS will cause what is called a “Curse of Dimensionality [10089]”. To solve this problem, it is necessary to extract the features of this dataset.

Feature extraction methods were combined with the characteristics of the data and the purpose of the study. Based on the high redundancy of gene sequencing and the need for diagnosis of SCZ, we assume the following:

1. The selected gene subset should be highly associated with whether the sample suffers from SCZ (the subset should be closely correlated with SCZ).
2. The correlation between each selected gene should be small (to eliminate the influence of redundant genes on diagnosis).

Based on the assumptions listed, we can obtain a subset of genes that contains feature genes with small redundancy that are highly correlated with SCZ. Machine learning, such as the CFS algorithm, has these same characteristics and is suitable for our study. It is widely used in studying other illnesses, such as cancer, and is reported to be an effective tool to analyze gene expression. Compared with other algorithms, CFS is quicker and more accurate in processing gene expression information [16]. After processing datasets with CFS, feature genes were selected, and a mathematical classifier was used to classify all samples, resulting in high sensitivity.

Takahashi et al.'s study [11] chose an unpaired *t*-test to analyze differentially regulated probes between two groups, evaluated every probe, and selected significantly differentially expressed genes (*P* < 0.01). By contrast, CFS deals with a subset of genes and considers the relationship between genes and genes with classification at the same time. This can ensure that each feature gene has a low correlation with other feature genes and high correlation with SCZ. Compared with Takahashi et al.'s study, we studied more samples and obtained fewer feature genes.

The interaction between genes is tremendously complicated. Statistical tests that make comparisons between the same genes, such as the paired *t*-test, might overlook the connection between genes. However, the CFS algorithm deals with a subset of genes, which considers both genes and interactions.

### 2.3. Feature Gene Selection

The core of the CFS algorithm is to evaluate a feature on its worth or merit [17, 18]. It considers the influence of features on predicting the class label together with the intercorrelation between each feature. The result of this algorithm is a subset which contains features highly correlated with the class and uncorrelated with each other.

This method calculates the relationship between each feature and class label (rcf) or, in other words, the relationship between gene expression and whether a person suffers SCZ. At the same time, it measures the intercorrelation between features (rff) and, in this study, the intercorrelation between genes:

(1) $$\begin{array}{c} Merit_{s}=\frac{k\overset{-}{\gamma_{cf}}}{\sqrt{k+k\left(k-1\right)\overset{-}{\gamma_{ff}}}}, \end{array}$$

where Merits is a feature subset *s* which contains *k* features, $\overset{-}{\gamma_{cf}}$ is the average correlation degree between features and categories, and $\overset{-}{\gamma_{ff}}$ is the average correlation degree between features. This method excludes genes unrelated to SCZ and the redundant genes highly correlated with one or more other genes. Equation (1) is a standard linear (Pearson's) correlation. Finally, 103 feature genes were selected, and a model was created.

Equation (1) was used for evaluating prediction performance of a certain set of genes. To obtain a gene set, we chose the “BEST-1st search” method. “BEST-1st search” is a search algorithm that explores a graph by expanding the most promising node according to a specified rule. By combining best-first-search with bidirectional-search, an ideal gene subset could be conveniently obtained.

### 2.4. Algorithm Application

Each sample in the merged dataset contains 11385 genes, and each gene is considered a property of a certain sample. The CFS algorithm is used to analyze every gene of every sample in the merged dataset. It then selects the genes with small redundancy and high correlation with SCZ. We extracted the selected genes in 290 samples and created a new dataset with only 103 feature genes.

The locally weighted learning (LWL) classifier saves all the data in the training set into memory and then calculates the distance between samples in the test set and the training set. Based on the calculated distance, LWL gives a higher weight to the training data which is closer to the test set. It then uses the weighted training set to learn and predict whether an unknown sample is a patient [19].

### 2.5. Pathway Analysis

To study biofunction of our results, 103 feature genes have been imported to the KOBAS website (http://kobas.cbi.pku.edu.cn/) We also performed KEGG pathway analysis (http://www.kegg.jp/kegg/pathway.html) to obtain biofunctional information about the feature genes.

## 3. Results

### 3.1. Feature Genes and Model

We analyzed 290 samples with 11385 genes. After normalization, 103 feature genes were selected by the CFS. All samples were used to create the model. It was tested by tenfold cross-validation and achieved 100% correct rate, which is higher than any other study's gene expression-based diagnostic, most of which vary from 70% to 100% [11, 20]. To diagnose whether a patient suffers from SCZ, the information from the patient's gene expression (Table S1) can be added to the existing datasets. Using the LWL classifier, which was the most effective dataset classifier in this study, we can know whether this person is an SCZ patient.

### 3.2. Validity of the Data Processing

For data-processing validation, this study used a comprehensive mathematical evaluation of the results.  Table 2 shows the results of 10-fold cross-validation on the dataset before and after CFS processing. Tenfold cross-validation is reported to be a useful tool for testing validity of result [21]. This analysis randomly divided the dataset into 10 groups, where 1 group was used for the test and the others were used for training. This is another widely used testing method. The result of assessment is listed in Table 2, and the result of evaluation in full training is listed in Table S2.

Compared with the unprocessed data, the processed data contains the same samples with a smaller number of genes (103 feature genes), avoiding the risk of falling into the “Curse of Dimensionality.” The selected gene subset is highly correlated with SCZ mathematically, and it excluded many redundant genes which would influence classification. Therefore, all the evaluation indicators were improved, reaffirming the validity of our data processing.

The CCI shows the percentage of instances of correct classification, directly describing the effectiveness of the classifier. RRSE represents the sum of absolute errors of n experiments and divides by the summation of the difference between actual value and average value. The lower this indicator is, the more accurate the classifier is. The *F*-measure is the harmonic mean of precision and recall. It is widely used in the field of IR (information retrieval) and is one of the crucial indicators to show the validity of a classifier. A good classifier's *F*-measure should be close to 1. The ROC area measures the area of ROC, and the PRC is the area below the correctly classified instances/all instances. A good classifier's ROC and PRC indicators should be close to 1.

**Figure 2:**
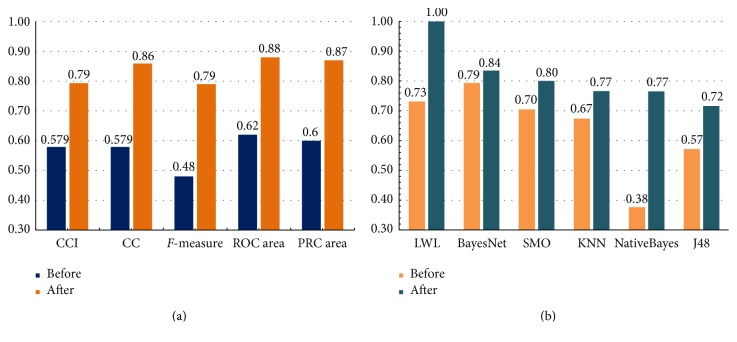
The comparisons of evaluation indicators before and after data processing. (a) Comparison of classifier training (NativeBayes) values. (b) Comparison of *F*-measure values.

After processing the data with the CFS algorithm, every evaluation indicator in the different classifier was improved. The LWL classifier shows the highest correct rate (100%) and is the ideal classifier in this model. This result indicates that our model is effective and reliable in the mathematical sense. From the table, it is clear that all evaluation indicators were improved after processing the original data, seen in Figures 2(a) and 2(b).

### 3.3. Pathway Analysis of 103 Feature Genes

After analysis, we obtained 9 pathways, which contained no less than 2 genes (*P* < 0.05). All the pathways selected have significant differences between the patient and control group (Table 3). Each of the pathways contains several input feature genes and some background genes. A lower *P* value means feature genes are more enriched in certain pathways.

## 4. Discussion

### 4.1. Effectiveness of Material and Normalization

We selected datasets from 4 groups of different schizophrenia patients to enlarge the sample size. At the same time, the normalization method we chose was consistent with the standard and proved effective [22]:

1. About XPN: Multidatasets across platform normalization results can maintain the highest interplatform concordance, but the number of samples contained in different, independent datasets should be similar.
2. About DWD: This method can make up for the deficiency of XPN. If there is a substantial difference between samples in different datasets, DWD could be used to normalize cross-platform datasets to reduce the loss of gene expression signals.

Finally, we obtained and studied a merged dataset which contains the largest sample size and the most features.

### 4.2. Biofunctional Verification

Biological studies verify the rationality of our feature genes distributed in pathways. For instance, the smallest *P* value is shown in Parkinson's disease. Parkinson's disease is reported to represent a strong, genetically defined level of comorbidity with schizophrenia [23]. Similarly, oxidative phosphorylation is enriched by feature genes we selected. The study of brain tissue from people with SCZ reveals that an oxidative phosphorylation defect caused metabolic disorders and is closely related to SCZ [24]. In addition, we predicted that TGF-beta has some connection with SCZ. Interestingly, research shows that the TGF-beta signaling pathway is highly associated with SCZ [25]. Overall, this suggests that feature genes are highly correlated with SCZ. Additionally, SCZ and Alzheimer's disease share the same molecular background [26]. Alzheimer's disease in our pathway analysis represents a relatively small *P* value. Additionally, primary immunodeficiency is an immune disorder and schizophrenia is correlated with the immune system [27]. From our results, the vascular smooth muscle contraction pathway should have some relationship with SCZ. Interestingly, it is one of the significant pathways in our prior study of SCZ [28].

Among 103 feature genes, 11 were found to be highly correlated with SCZ in previous research. Low functioning Asn107 variant NPSR1 causes a disorder of the neuropeptide S (NPS) neurotransmitter system. NPSR1 is identified to be associated with SCZ [29]. Additionally, NPSR1 is one of the feature genes separated by the CFS algorithm. Compared with normal controls, a lower level of expression of SLC3A2 in peripheral white blood cell is shown in people with schizophrenia [30]. Patients with SCZ show elevated TSPO binding in PET in vivo brain imaging [31]. These studies indicate that, at the genetic level, some of our feature genes' relationships with SCZ is demonstrated biologically by previous research.

### 4.3. Pathway Distribution

**Figure 3:**
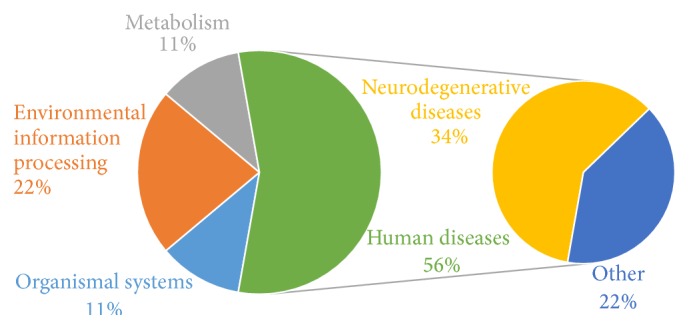
Functional category by pathways.

We performed bioinformatics analysis of feature genes, and the result is shown in Figure 3. Of the 56% of pathways that are found in human diseases, neurodegenerative diseases make up 34%. This shows a strong connection between SCZ and degeneration of nerves. Environmental information processing, which contains 22% of all pathways, also plays a role in SCZ. Metabolism and organismal systems show some influence on the pathology of schizophrenia.

The enrichment of neurodegenerative disease-related feature genes provides supporting evidence for the role of neurodegenerative dysfunction in schizophrenia [32]. Recently, a study on mice showed that SCZ is associated with a disorder in environmental information interaction, which is influenced by environmental information processing [33].

## 5. Conclusion

Using mathematical and biological verification to examine whether the CFS-LWL algorithm is an effective method to distinguish people with SCZ from normal controls, and we find the superiority of CFS-LWL algorithm in testing whether a sample is an SCZ patient:

1. The correlation-based feature selection (CFS) algorithm was proposed and a model for SCZ diagnosis was built. The whole blood gene expression data, including 152 individuals with schizophrenia (SCZ) and 138 normal controls, were analyzed based on CFS.
2. 103 significantly differentially feature genes were selected from the random 10 groups of samples, and the feature genes were enriched in Parkinson's disease, oxidative phosphorylation, and TGF-beta signaling pathways, which were previously reported to be associated with SCZ.
3. The cross-validation showed that the model we constructed achieved nearly 100% classification accuracy. The mathematical evaluation of the datasets before and after data processing proved the effectiveness of our algorithm.

## Supplementary Material

Table S1 lists the top 100 feature genes by CFS for SCZ. Table S2 lists the evaluation result of training all set by CFS.

## Figures and Tables

**Table 1:** Detailed information about dataset collected.

Dataset	Case	Control	Tissue	Platform
*GSE54913 *	18	12	Blood	GPL15314 Arraystar Human LncRNA microarray V2.0 (Agilent_033010 Probe Name version)
*GSE38481 *	15	22	Blood	GPL6883 Illumina HumanRef-8 v3.0 expression beadchip
*GSE38484 *	106	96	Blood	GPL6947 Illumina HumanHT-12 V3.0 expression beadchip
*GSE18312 *	13	8	Blood	GPL5175 [HuEx-1_0-st] Affymetrix Human Exon 1.0 ST Array

**Table 2:** The evaluation results of data processing.

	LWL		BayesNet		SMO		KNN		NativeBayes		J48	
	Before	After	Before	After	Before	After	Before	After	Before	After	Before	After
CCI^*∗*^ (%)	73.1	**100.0**	79.3	**83.4**	72.4	**80.0**	69.0	**76.6**	51.7	**76.6**	58.6	**71.7**
RRSE^*∗*^ (%)	85.8	**16.6**	91.3	**77.5**	105.4	**89.5**	81.8	**85.4**	139.4	**93.8**	128.0	**103.5**
CC^*∗*^ (%)	99.7	**100.0**	79.3	**89.0**	72.4	**80.0**	100.0	**95.5**	51.7	**83.1**	62.1	**77.9**
*F*-measure	0.73	**1.00**	0.80	**0.83**	0.71	**0.80**	0.67	**0.76**	0.37	**0.76**	0.57	**0.71**
ROC^*∗*^ area	0.81	**1.00**	0.83	**0.92**	0.70	**0.80**	0.84	**0.81**	0.48	**0.82**	0.51	**0.72**
PRC^*∗*^ area	0.80	**1.00**	0.79	**0.92**	0.66	**0.74**	0.84	**0.79**	0.49	**0.81**	0.53	**0.67**

^*∗*^CCI, correctly classified instances; RRSE, Root Relative Squared Error; CC, Coverage of Cases (0.95 level). ROC, Receiver Operating Characteristic; PRC, Precision and Recall Curve.

**Table 3:** The pathway results of significant differences between patient and control group.

Feature	ID	Input number	Background number	*P* value
Parkinson's disease	hsa05012	6	108	7.96*E* − 05
Oxidative phosphorylation	hsa00190	4	98	0.003793377
TGF-beta signaling pathway	hsa04350	3	66	0.009126485
Metabolic pathways	hsa01100	14	1118	0.010961336
Alzheimer's disease	hsa05010	4	139	0.012275212
Primary immunodeficiency	hsa05340	2	34	0.021039942
Huntington's disease	hsa05016	4	165	0.021326367
Vascular smooth muscle contraction	hsa04270	3	109	0.032774232
ABC transporters	hsa02010	2	44	0.033204998
Nonalcoholic fatty liver disease	hsa04932	3	129	0.049322146

Note: the KEGG pathway analysis shows genes in these pathways are significantly differently expressed (*P* < 0.05). In addition, each pathway contains no fewer than 2 genes.
