# Supplementary material for: The Correlation-Base-Selection Algorithm for Diagnostic Schizophrenia Based on Blood-Based Gene Expression Signatures
Source: Biomed Res Int. 2017 Feb 9;2017:7860506. doi: 10.1155/2017/7860506 (PMC5322573; doi:10.1155/2017/7860506)
Supplement: Supplementary file 1 — Table S1 lists the top 100 feature genes by CFS for SCZ. Table S2 lists the evaluation result of training all set by CFS. [file 7860506.f1.docx]

**Table S1** Feature genes selected by CFS.

| GENE ID | GENE SYMBOL | GENE NAME |
| --- | --- | --- |
| 95 | ACY1 | aminoacylase 1 |
| 268 | AMH | anti-Mullerian hormone |
| 471 | ATIC | 5-aminoimidazole-4-carboxamide ribonucleotide formyltransferase/IMP cyclohydrolase |
| 509 | ATP5C1 | ATP synthase, H+ transporting, mitochondrial F1 complex, gamma polypeptide 1 |
| 566 | AZU1 | azurocidin 1 |
| 706 | TSPO | translocator protein |
| 959 | CD40LG | CD40 ligand |
| 1534 | CYB561 | cytochrome b561 |
| 1634 | DCN | decorin |
| 1669 | DEFA4 | defensin alpha 4 |
| 1847 | DUSP5 | dual specificity phosphatase 5 |
| 2002 | ELK1 | ELK1, member of ETS oncogene family |
| 2257 | FGF12 | fibroblast growth factor 12 |
| 2302 | FOXJ1 | forkhead box J1 |
| 2517 | FUCA1 | fucosidase, alpha-L- 1, tissue |
| 2583 | B4GALNT1 | beta-1,4-N-acetyl-galactosaminyl transferase 1 |
| 2745 | GLRX | glutaredoxin |
| 2992 | GYG1 | glycogenin 1 |
| 3084 | NRG1 | neuregulin 1 |
| 3212 | HOXB2 | homeobox B2 |
| 3671 | ISLR | immunoglobulin superfamily containing leucine-rich repeat |
| 3778 | KCNMA1 | potassium channel, calcium activated large conductance subfamily M alpha, member 1 |
| 4697 | NDUFA4 | NDUFA4, mitochondrial complex associated |
| 4857 | NOVA1 | neuro-oncological ventral antigen 1 |
| 5095 | PCCA | propionyl-CoA carboxylase alpha subunit |
| 5216 | PFN1 | profilin 1 |
| 5326 | PLAGL2 | PLAG1 like zinc finger 2 |
| 5413 | SEPT5 | septin 5 |
| 5496 | PPM1G | protein phosphatase, Mg2+/Mn2+ dependent 1G |
| 5498 | PPOX | protoporphyrinogen oxidase |
| 6175 | RPLP0 | ribosomal protein, large, P0 |
| 6451 | SH3BGRL | SH3 domain binding glutamate-rich protein like |
| 6520 | SLC3A2 | solute carrier family 3 (amino acid transporter heavy chain), member 2 |
| 6619 | SNAPC3 | small nuclear RNA activating complex polypeptide 3 |
| 6888 | TALDO1 | transaldolase 1 |
| 6890 | TAP1 | transporter 1, ATP-binding cassette, sub-family B (MDR/TAP) |
| 6897 | TARS | threonyl-tRNA synthetase |
| 7568 | ZNF20 | zinc finger protein 20 |
| 7866 | IFRD2 | interferon-related developmental regulator 2 |
| 8233 | ZRSR2 | zinc finger (CCCH type), RNA binding motif and serine/arginine rich 2 |
| 8399 | PLA2G10 | phospholipase A2 group X |
| 8454 | CUL1 | cullin 1 |
| 9230 | RAB11B | RAB11B, member RAS oncogene family |
| 9270 | ITGB1BP1 | integrin subunit beta 1 binding protein 1 |
| 9315 | NREP | neuronal regeneration related protein |
| 9349 | RPL23 | ribosomal protein L23 |
| 9556 | C14orf2 | chromosome 14 open reading frame 2 |
| 9557 | CHD1L | chromodomain helicase DNA binding protein 1-like |
| 9647 | PPM1F | protein phosphatase, Mg2+/Mn2+ dependent 1F |
| 9805 | SCRN1 | secernin 1 |
| 9975 | NR1D2 | nuclear receptor subfamily 1 group D member 2 |
| 9978 | RBX1 | ring-box 1, E3 ubiquitin protein ligase |
| 10228 | STX6 | syntaxin 6 |
| 10474 | TADA3 | transcriptional adaptor 3 |
| 10765 | KDM5B | lysine (K)-specific demethylase 5B |
| 10917 | BTNL3 | butyrophilin-like 3 |
| 11011 | TLK2 | tousled like kinase 2 |
| 11070 | TMEM115 | transmembrane protein 115 |
| 11119 | BTN3A1 | butyrophilin subfamily 3 member A1 |
| 11315 | PARK7 | parkinson protein 7 |
| 22823 | MTF2 | metal response element binding transcription factor 2 |
| 22976 | PAXIP1 | PAX interacting (with transcription-activation domain) protein 1 |
| 23135 | KDM6B | lysine (K)-specific demethylase 6B |
| 23199 | GSE1 | Gse1 coiled-coil protein |
| 23309 | SIN3B | SIN3 transcription regulator family member B |
| 25976 | TIPARP | TCDD-inducible poly(ADP-ribose) polymerase |
| 27304 | MOCS3 | molybdenum cofactor synthesis 3 |
| 51023 | MRPS18C | mitochondrial ribosomal protein S18C |
| 51102 | MECR | mitochondrial trans-2-enoyl-CoA reductase |
| 51160 | VPS28 | vacuolar protein sorting 28 homolog (S. cerevisiae) |
| 51651 | PTRH2 | peptidyl-tRNA hydrolase 2 |
| 51673 | TPPP3 | tubulin polymerization-promoting protein family member 3 |
| 54058 | C21orf58 | chromosome 21 open reading frame 58 |
| 54807 | ZNF586 | zinc finger protein 586 |
| 54838 | WBP1L | WW domain binding protein 1-like |
| 55022 | PID1 | phosphotyrosine interaction domain containing 1 |
| 55754 | TMEM30A | transmembrane protein 30A |
| 55967 | NDUFA12 | NADH:ubiquinone oxidoreductase subunit A12 |
| 56052 | ALG1 | ALG1, chitobiosyldiphosphodolichol beta-mannosyltransferase |
| 56961 | SHD | Src homology 2 domain containing transforming protein D |
| 57470 | LRRC47 | leucine rich repeat containing 47 |
| 57634 | EP400 | E1A binding protein p400 |
| 79171 | RBM42 | RNA binding motif protein 42 |
| 79650 | USB1 | U6 snRNA biogenesis 1 |
| 83743 | GRWD1 | glutamate-rich WD repeat containing 1 |
| 84232 | MAF1 | MAF1 homolog, negative regulator of RNA polymerase III |
| 84314 | TMEM107 | transmembrane protein 107 |
| 85414 | SLC45A3 | solute carrier family 45 member 3 |
| 93621 | MRFAP1 | Morf4 family associated protein 1 |
| 94015 | TTYH2 | tweety family member 2 |
| 94160 | ABCC12 | ATP binding cassette subfamily C member 12 |
| 94274 | PPP1R14A | protein phosphatase 1 regulatory inhibitor subunit 14A |
| 115825 | WDFY2 | WD repeat and FYVE domain containing 2 |
| 125965 | COX6B2 | cytochrome c oxidase subunit VIb polypeptide 2 (testis) |
| 128653 | C20orf141 | chromosome 20 open reading frame 141 |
| 131669 | UROC1 | urocanate hydratase 1 |
| 134510 | UBLCP1 | ubiquitin like domain containing CTD phosphatase 1 |
| 145389 | SLC38A6 | solute carrier family 38 member 6 |
| 147657 | ZNF480 | zinc finger protein 480 |
| 147912 | SIX5 | SIX homeobox 5 |
| 221188 | GPR114 | adhesion G protein-coupled receptor G5 |
| 375061 | FAM89A | family with sequence similarity 89 member A |
| 387129 | NPSR1 | neuropeptide S receptor 1 |
