# Supplementary material for: The Correlation-Base-Selection Algorithm for Diagnostic Schizophrenia Based on Blood-Based Gene Expression Signatures
Source: Biomed Res Int. 2017 Feb 9;2017:7860506. doi: 10.1155/2017/7860506 (PMC5322573; doi:10.1155/2017/7860506)
Supplement: Supplementary file 2 [file 7860506.f2.docx]

**Table S2.** The result of evaluation in full training set.

|  | **LWL** | | **BayesNet** | | **SMO** | | **KNN** | | **NativeBayes** | | **J48** | |
| --- | --- | --- | --- | --- | --- | --- | --- | --- | --- | --- | --- | --- |
|  | before | after | before | after | before | after | before | after | before | after | before | after |
| (%) | 100.0 | **100.0** | 75.5 | **91.7** | 74.1 | **81.4** | 73.4 | **81.7** | 57.9 | **79.3** | 98.3 | **98.3** |
| (%) | 22.4 | **16.6** | 98.7 | **50.4** | 101.8 | **86.4** | 82.1 | **73.4** | 129.9 | **84.4** | 24.2 | **23.6** |
| (%) | 100.0 | **100.0** | 75.9 | **95.9** | 74.1 | **81.4** | 100.0 | **100.0** | 57.9 | **85.9** | 99.7 | **99.7** |
| F-measure | 1.00 | **1.00** | 0.75 | **0.92** | 0.74 | **0.81** | 0.73 | **0.82** | 0.48 | **0.79** | 0.98 | **0.98** |
| ROC area | 1.00 | **1.00** | 0.84 | **0.98** | 0.74 | **0.81** | 0.84 | **0.90** | 0.62 | **0.88** | 1.00 | **0.99** |
| PRC area | 1.00 | **1.00** | 0.78 | **0.98** | 0.68 | **0.76** | 0.83 | **0.89** | 0.60 | **0.87** | 1.00 | **0.99** |
| 1.Correctly Classified Instances | | | | |  |  |  |  |  |  |  |  |
| 2.Root relative squared error | | | | |  |  |  |  |  |  |  |  |
| 3.Coverage of cases (0.95 level) | | | | |  |  |  |  |  |  |  |  |
